# Microbiome of Co-cultured Fish Exhibits Host Selection and Niche Differentiation at the Organ Scale

**DOI:** 10.3389/fmicb.2019.02576

**Published:** 2019-11-08

**Authors:** Zhimin Zhang, Dapeng Li, Weitong Xu, Rong Tang, Li Li

**Affiliations:** ^1^Hubei Provincial Engineering Laboratory for Pond Aquaculture, College of Fisheries, Huazhong Agricultural University, Wuhan, China; ^2^Institute of Hydrobiology, Chinese Academy of Sciences, Wuhan, China

**Keywords:** grass carp (*Ctenopharyngodon idella*), southern catfish (*Silurus meridionalis*), microbial community, gastrointestinal tract, skin, gill

## Abstract

Fish are the most widespread aquaculture species and maintain complex associations with microbial consortiums. However, the ecology of these associations present in multiple microhabitats in fish remains elusive, especially on the microbial assembly in fish external (skin and gill) and internal (stomach and intestine) niches, and the relationship with the rearing environment. To understand host dependence and niche differentiation of organ-specific microbiome signatures using a 16S rRNA gene-based sequencing technique, we systematically provided characterizations of a comparative framework relevant to the microbiome of stomach, regional intestine, skin, and gill in two important farmed fish species, herbivorous grass carp (*Ctenopharyngodon idella*) and carnivorous southern catfish (*Silurus meridionalis*), and of the rearing water. The different feeding habits of grass carp and southern catfish showed a significant separation of microbial community structure, with great compositional differences across body sites within each species. Site-driven divergences relied on host species: the same types of microhabitats between grass carp and southern catfish harbored differential microbiome. Additionally, body sites had remarkably distinct communities and displayed lower alpha diversity compared to rearing water. Unexpectedly, the stomach of southern catfish had the highest microbial diversity in the digestive tract of the two co-cultured fish species. For external sites within each species, a higher diversity occurred in gill of grass carp and in skin of southern catfish. Our results unveil different topographical microbiome signatures of the co-cultured species, indicating host selection in individual-level microbial assemblages and niche differentiation at the organ scale. This work represents a foundation for understanding the comprehensive microbial ecology of cohabiting farmed fish, suggesting potential applications associated with fish microbiome that urgently needs to be assessed in polycultured operations in aquaculture.

## Introduction

Hosts naturally harbor complex microbial population resident in multiple body habitats. The assembly and function of host-microbiota symbiosis are central to host nutrition, development of mucosal layer, homeostasis of the immune system, and resistance to pathogenic taxa (Lee and Mazmanian, 2010; Nicholson et al., 2012; Gomez et al., 2013; Banerjee and Ray, 2017). Since the initials of Human and Health and Microbiome, studies on the overall ecology of host microbiome have focused on human in relation to gut, mouth, skin, and vaginal cavity (Nicholson et al., 2012; Romero et al., 2014; Yildirim et al., 2014; SanMiguel and Grice, 2015; Proctor and Relman, 2017). Gut is the most extensively studied anatomical site for microbial ecology with the advance of high-throughput sequencing. Indeed, it is an ideal reservoir for microbial colonization in humans (Lee and Mazmanian, 2010; Nicholson et al., 2012) and also in numerous terrestrial and aquatic vertebrates (Pascoe et al., 2017). The characterizations of gut microbiome greatly depend on specific groups of organisms. In natural populations, besides host-related factors such as sex and age (Bolnick et al., 2014; Jašarević et al., 2017), local environments such as pH and temperature contribute to gut microbial variations (Amato et al., 2013; Bolnick et al., 2014; Kohl and Yahn, 2016). The effects of geographic habitats have been reported on the microbial community of specific hosts like humans, rodents, poultry, and fish (Bolnick et al., 2014; Zhang et al., 2015; Pascoe et al., 2017). However, a major challenge to our understanding of microbial ecology in animals is that it is rather difficult to unravel one effect from the other due to highly variable geographic environments and host complexity.

Aquatic environments are changeful habitats for many vertebrates, and extensive studies are now going into investigating and cataloging microbial ecology in fish, especially the model organism zebrafish (Bates et al., 2006; Kanther and Rawls, 2010; Stagaman et al., 2017) and some commercial species such as rainbow trout (*Oncorhynchus mykiss*; Lowrey et al., 2015), Atlantic salmon (*Salmo salar*; Green et al., 2013; Gajardo et al., 2016; Schmidt et al., 2016; Zarkasi et al., 2016; Reid et al., 2017), southern catfish (*Silurus meridionalis*; Zhang et al., 2017), and several carps (Hao et al., 2017; Tran et al., 2017). On one hand, different aquatic environments could be associated with radical shifts in feed sources and ecological spectrum for fish; on the other hand, poikilothermic fish respond quickly to external perturbations. Aquatic environment is an ideal medium for the growth and spread of microbial community that could affect individual development and health *via* cross talk and various interactions at mucosal surfaces. Germ-free environments make zebrafish (*Danio rerio*) larvae shape a degenerative phenotype, eventually leading to a failure of protein macromolecules digestion (Bates et al., 2006). This suggests the importance of microbial interactions with hosts. In addition, hydrological shifts in aquatic environments could result in host microbial divergence (Amato et al., 2013; Bolnick et al., 2014; Kohl and Yahn, 2016; Sylvain et al., 2016), indicating the possibility of restructuring microbial communities in external organs, such as skin and gill. Despite a plethora of studies on internal fish microbiome available (Bolnick et al., 2014; Llewellyn et al., 2014; Lowrey et al., 2015; Sylvain et al., 2016; Zarkasi et al., 2016; Tarnecki et al., 2017; Tran et al., 2017; Zhang et al., 2017), literature is primitive on the knowledge of the external microbiome.

To maximize utilizations of feed and space sources depending on different characterizations of ecological niches of fish species, it is a common practice in aquaculture to farm multiple fish species in the same water body, called polyculture. It is a combination of different species that are living in different niches of the pond in order to utilize the resources (e.g., feed resources, space) more efficiently without competing with each other. Co-cultured species might have evolutionarily formed different traits, such as the tolerance on dissolved oxygen and temperature, and disease susceptibility. The adaptation of fish to surrounding environments and applied feeds is directly related to their biological organs. Like differential internal microbiome reported in fish (Ye et al., 2014; Gajardo et al., 2016; Zhang et al., 2017), it could be suspected that external microbiome is also diversely shaped.

The fastest growing sector in agriculture is aquaculture. Grass carp, *Ctenopharyngodon idella*, is the most important contributor to fish production globally (Fisheries and Aquaculture Department [FAO], 2018). In practice, it is often cultured with various kinds of fish species, such as southern catfish, because it is a benthic fish and feeds on small fish and/or residual feeds in polyculture systems to efficiently improve feed and space utilizations. Grass carp and southern catfish have significantly different properties: the former is a scaled, herbivorous fish with long intestine, and the latter is a scaleless, carnivorous fish with a stomach. The diverse characteristics determine the structural and functional differences in multiple organs, such as skin and digestive tract, and different potentials for microbial colonization on the organs. Therefore, we compared the microbiome signatures among the two fish species and their environmental surrounding and further hypothesized host-mediated impacts on fish microbiome and niche differentiation of the microbiome at the organ scale.

## Materials and Methods

### Experimental Setup and Sample Collection

**FIGURE 1:**
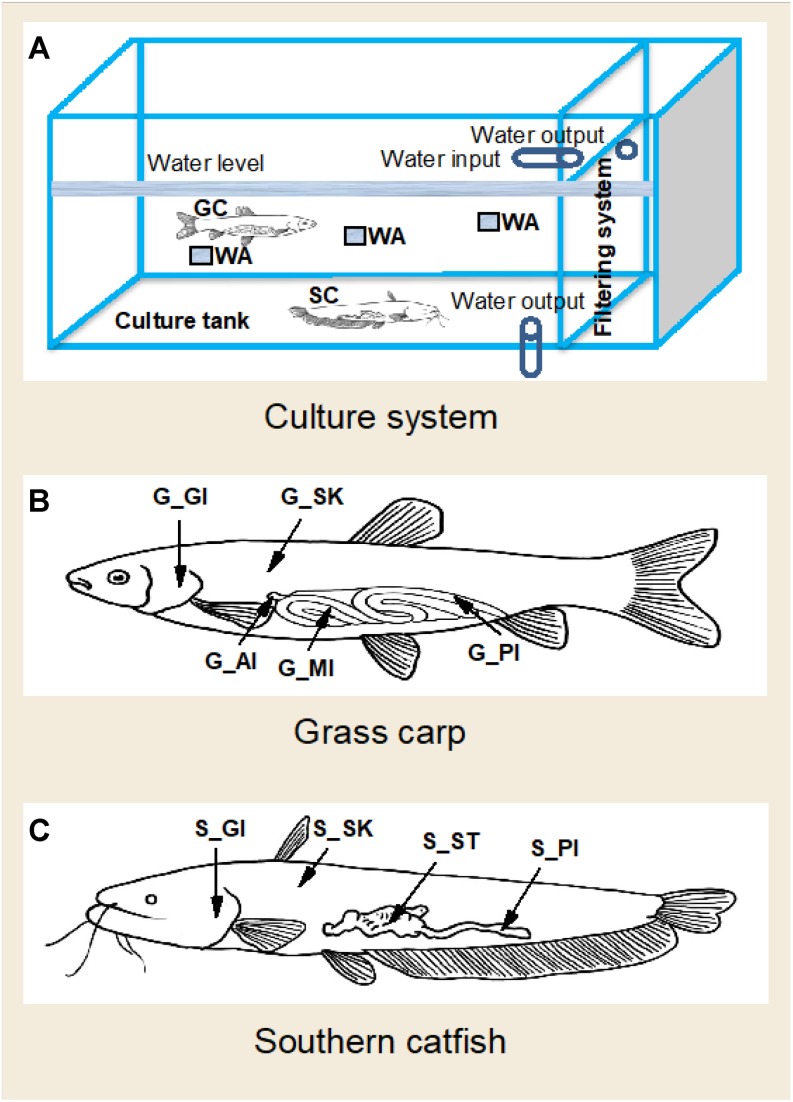
A diagram of culture system and sampling strategy of the organs in co-cultured grass carp and southern catfish and of their rearing water. GC, grass carp; SC, southern catfish; WA, rearing water; G-AI, anterior intestine of grass carp; G-MI, medium intestine of grass carp; G-PI, posterior intestine of grass carp; G-GI, gill of grass carp; G-SK, skin of grass carp; S-ST, stomach of southern catfish; S-PI, posterior intestine of southern catfish; S-GI, gill of southern catfish; S-SK, skin of southern catfish. **(A)** Culture system; **(B)** Grass carp; **(C)** Southern catfish.

All data were generated from a laboratory study performed at the College of Fisheries, Huazhong Agricultural University. Grass carp and southern catfish were stocked in separate tanks for at least 2 months for experimental acclimatization before the experiment. At the start of the experiment, 24 grass carp and 16 catfish were transferred into a tank with circulation system. The effective tank size for fish was 2.1 × 1.2 × 0.55 m, and the filtering unit comprised mechanical filters, ceramic ring biofilters, and abstract brush (Figure 1). During the experimental period, the two fish species were hand-fed until satiety with a commercial high-protein feed (K203) from Wuhan Coland Feed Co., Ltd., and water renewal was 30% each 3 days. Recent studies have reported that dietary interventions alter gut microbiota, which tends to be stable within a week (Hao et al., 2017), and that 2 weeks of transfer experiments at different aquatic environments reveal changes in microbial community of host skin (Bletz et al., 2016). In this study, to make fish assemble stable microbial profiles, the experiment was extended and designed to have a short interval to explore whether microbial communities of the two fish species are relatively stable. We collected samples at 42 days and 45 days of the experiment for temporal microbial analysis. In order to obtain the background composition of environmental microbiome, four commercial feed samples and six rearing water samples were collected. However, no microbe was detected in the feed. It may be attributed to high temperature and germicidal treatments of the feed production. At each sampling date, dissolved oxygen, pH, and temperature of rearing water were measured using Thermo Scientific, ORION STRA A221 pH meter. Moreover, three water samples with each 500 ml in volume were collected in different depths, below the surface water about 10, 20, and 30 cm, as shown in Figure 1A. They were filtered through 0.22-μm glass fiber filters for microbial analysis. The filtered water was used for chemical analysis (Supplementary Table S1). To avoid the potential effects of anesthetic use on fish skin and gill microbiome, the method of percussive stunning was used before sampling in this study. Body weight and length of the fish were measured (Supplementary Table S2). Four grass carp and five southern catfish were dissected under sterile conditions at each time event. Skin (scale and mucus), gill, anterior intestine and posterior intestine were collected from grass carp; meanwhile, skin (mucus), gill, stomach, and posterior intestine of southern catfish were sampled. All procedures were performed in accordance with international guidelines and regulations for the use of animals in research. The study was approved by the Scientific Ethics Committee of Huazhong Agricultural University under permit number HZAUFI-2016-008. The sampling scheme was described in Figures 1B,C. All of the microbial samples collected were immediately frozen at −80°C for microbial analysis.

### DNA Extraction and 16S rRNA Gene PCR Amplification

Genomic DNA was extracted from the fish and feed samples using QIAamp DNA Stool Mini Kit (Qiagen, Hilden, NRW, Germany) and from the water samples using Mo PowerWater DNA Isolation kits (MoBio, Valencia, CA, United States) following the manufacturer’s protocol with minor modifications to increase DNA yield, including resuspension in 100 μl TE buffer solution. The V4-V5 region of the bacteria was PCR-amplified with the bar coded-primers (515F, 5′-GTGCCAGCMGCCGCGGTAA-3′ and 907R, 5′-CCG TCAATTCCTTTGAGTTT-3′). PCR reactions consisted of 50 μl mixtures with 100 ng DNA templates, 0.4 μM forward and reverse primers, 2.5 U of GoTaq Flexi Polymerase (Promega, United States), 200 μM deoxynucleoside triphosphate (dNTP), and 2 mM MgCl_2_. Each sample was amplified in duplicate PCR, and then the PCR products were combined into one final pool in equal concentration. PCR conditions were: a denaturation step of 94°C for 5 min, followed by 25 cycles at 94°C for 30 s, 55°C for 30 s, 72°C for 60 s, and a final extension at 72°C for 5 min. Unique combinations of all used forward and reverse primers as negative control and one positive control with water as a template were carried out at each 96-well plate. PCR products were purified using Qiagen Gel Extraction Kit according to the manufacturer’s instructions (Qiagen, Germany) and then quantified using PicoGreen reagent. The paired-end sequencing was performed on an Illumina HiSeq platform (HiSeq Reagent Kit V.2, 500 cycles).

### Sequence Processing and Statistical Analysis

Quantitative Insights Into Microbial Ecology (QIIME v1.7.0) with default settings was used for all the 16S rRNA sequence analyses. The sequence reads were filtered, and the chimeric sequences were removed using UCHIME algorithm prior to pick the operational taxonomic units (OTUs), with CD-HIT method based on 97% identity to entries in the Greengenes database (v13_8). OTUs with singletons were filtered out, and the RDP classifier was used to assign microbial taxonomy with an 80% confidence threshold. Eventually, all samples contained a total of 7468265 sequences (range: 29141–314884 sequences per sample; average: 86840). Raw data sets are available at the Sequence Read Archive of NCBI under accession number PRJNA518052. Analyses were performed on data rarefied to 27400 sequences per sample to allow inclusion of all of the samples and avoid bias of microbial diversity present within samples due to different sequence depths.

Both alpha and beta diversity indices of microbial community of all samples were calculated using QIIME. We calculated alpha diversity using four indices: Shannon diversity, observed species, Chao1, and ACE. Before testing differences in alpha-diversity among body sites, we used a Shapiro–Wilks test to verify homogeneity of variance. When data could meet equal variances, we used *t*-test to test differences between groups and one-way analysis of variance (ANOVA) for more groups; otherwise, Welch’s *t*-test for two groups and Kruskal–Wallis test for more groups were used in IBM SPSS Statistics 21. Beta diversity was calculated using three distance matrices algorithms: Bray-Curtis, unweighted UniFrac, and weighted UniFrac. The full data sets or the subsampled data sets were visualized using non-metric multidimensional scaling (NMDS) and principal coordinates analysis (PCoA) with the ggplot2 package in R version 3.0.2. We further used analysis of similarities (ANOSIM) and non-parametric permutational multivariate analysis of variance (PERMANOVA) of 999 permutations with vegan package to evaluate the microbial differences. For all tests, *P* ≤ 0.05 was considered a statistically significant difference.

## Results

### Temporal Coherence of Microbiome in Cohabiting Fish and the Rearing Water

**FIGURE 2:**
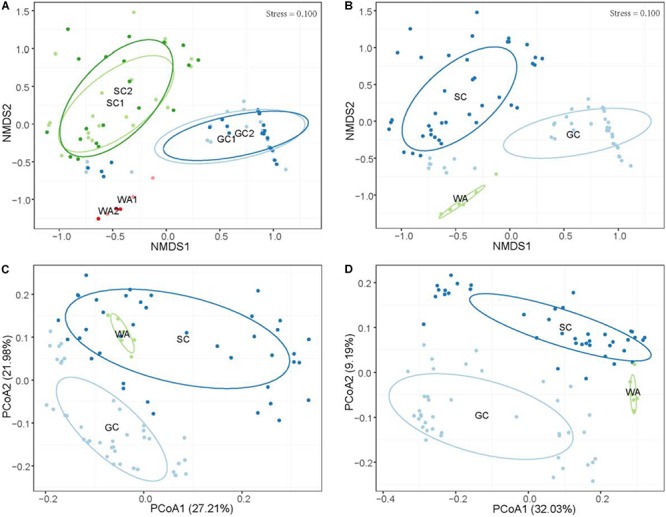
Differential microbiome communities of grass carp, southern catfish, and their rearing water. **(A)** Non-metric multidimensional scaling (NMDS) based on Bray-Curtis distance metrics of microbial communities of all organs from grass carp and southern catfish and their rearing water at 42 and 45 days. **(B)** NMDS based on Bray-Curtis distance metrics of microbial communities of all organs from grass carp and southern catfish and their rearing water during the whole experimental period; PCoA based on weighted **(C)** and unweighted **(D)** UniFrac distance metrics of microbial communities of all organs from grass carp and southern catfish and their rearing water. GC, grass carp; SC, southern catfish; WA, rearing water; GC1, grass carp collected at 42 days; GC2, grass carp collected at 45 days; SC1, southern catfish collected at 42 days; SC2, southern catfish collected at 45 days; WA1 and WA2 represent rearing water collected at 42 and 45 days, respectively.

The results showed that there were no clear perturbations on water parameters at the two sampling times (Supplementary Table S1). Meanwhile, no temporal differences were observed in the microbiome community of grass carp (PERMANOVA, *P* > 0.05) and southern catfish (PERMANOVA, *P* > 0.05) as well as their rearing water (PERMANOVA, *P* > 0.05) based on Bray-Curtis distances, as shown by the clear overlaps within each host or the environment sample sources (Figure 2A). This indicates that in this study the microbiome of fish in the controlled conditions had relatively stable configurations of microbiome. Therefore, we incorporated the same type of samples collected from each species at the two time points in subsequent analyses.

### Host-Mediated and Environmental Microbiome Community

**FIGURE 3:**
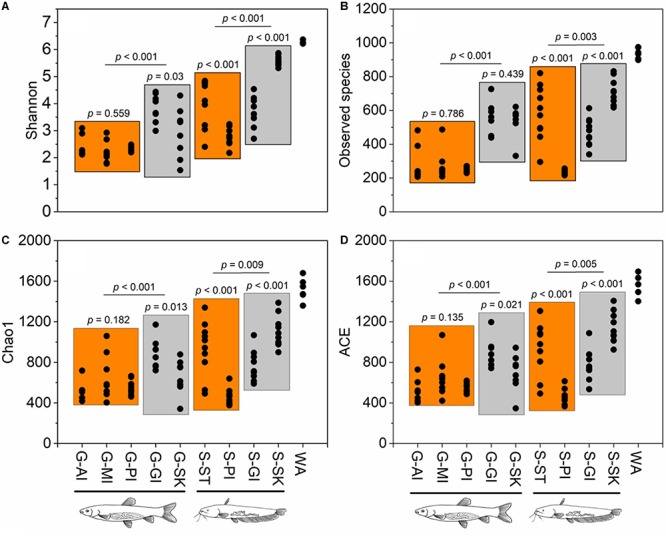
Alpha diversity estimates across sample types of co-cultured southern catfish and grass carp and rearing water. Four alpha diversity indices show similar patterns of variations in microbial diversity within or between sample types. **(A)** Shannon index; **(B)** observed species; **(C)** Chao 1; **(D)** ACE.

To investigate host effects on fish microbiome and differences in microbiome between fish and their environment, we simultaneously used Bray-Curtis, weighted UniFrac, and unweighted UniFrac distance matrices to quantify microbial communities using PEMANOVA analysis. Significant differences in the microbiome were found among two fish species and rearing water (Bray-Curtis, *P* < 0.001, Figure 2B; weighted UniFrac, *P* < 0.001, Figure 2C; unweighted UniFrac, *P* < 0.001, Figure 2D). The pairwise comparisons of PERMANOVA showed significantly different microbiomes between the fish and rearing water (grass carp vs. water, *P* < 0.001 for all distances; southern catfish vs. water, *P* < 0.001 for all distances). In addition, we found significantly different alpha diversities among the cohabiting fish and water (Shannon diversity, observed species, Chao1, and ACE; Kruskal–Wallis test, *P* < 0.001 in all cases). Moreover, the highest level of alpha diversity was found in the rearing water of the cohabiting fish (Figure 3).

### Differences in Microbiome Community Across Body Sites

**FIGURE 4:**
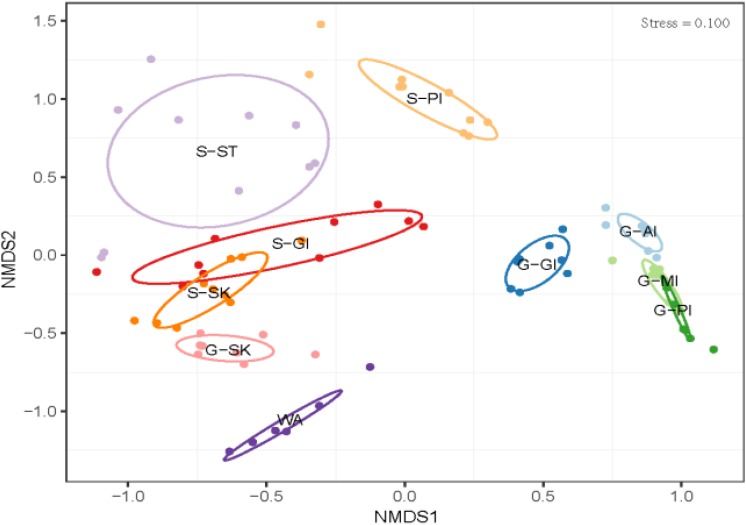
Microbial community differences in co-cultured grass carp and southern catfish at the organ scale and their rearing water. WA, rearing water; G-AI, anterior intestine of grass carp; G-MI, medium intestine of grass carp; G-PI, posterior intestine of grass carp; G-GI, gill of grass carp; G-SK, skin of grass carp; S-ST, stomach of southern catfish; S-PI, posterior intestine of southern catfish; S-GI, gill of southern catfish; S-SK, skin of southern catfish.

The results showed differentiations in body site-associated microbiome in grass carp and southern catfish (Figure 4). In order to further quantify sources of the differences in microbiome communities in the cohabiting grass carp and southern catfish, a subsample of the data set that included two fish species or each fish species was used for microbial analysis. The fish microbiome was significantly affected not only by fish species (PERMANOVA, Bray-Curtis, *R*^2^ = 0.2735, *P* = 0.001) but also by body sites (PERMANOVA, Bray-Curtis, *R*^2^ = 0.37295, *P* = 0.001) (Supplementary Table S3). The analyses of overall compositions by ANOSIM tests showed high microbiome variations among body sites in grass carp (ANOSIM, *R* = 0.874, *P* = 0.001) and southern catfish (ANOSIM, *R* = 0.899, *P* = 0.001). In pairwise *a posteriori* tests of PERMANOVA, the microbiome communities in any two body sites were statistically different (Table 1). The communities between external body sites were less similar than those between internal body sites. The microbiome between the same type of body sites (external or internal) was generally more similar than that between the different types of body sites (external and internal). In southern catfish, external microbiome within specific body sites had higher interindividual variability than their internal microbiome (*P* < 0.001). Compared to southern catfish, grass carp had the smaller interindividual dissimilarities of the microbiome among body sites (*P* < 0.001) and that were comparable among the organs (*P* > 0.05) except for anterior intestine.

**TABLE 1:** Body site-driven microbiome differences in co-cultured grass carp and southern catfish.

**Fish species**	**Organs**	**Adonis**		
		***F*-Model**	***R*^2^**	***P*-value**
Grass carp	AI vs. MI	24.6983	0.6382	0.001
	AI vs. PI	31.3769	0.6915	0.001
	MI vs. PI	5.368	0.2772	0.022
	AI vs. GI	8.1625	0.3683	0.002
	AI vs. SK	198.334	0.934	0.001
	MI vs. GI	48.9677	0.7776	0.002
	MI vs. SK	326.5514	0.9588	0.001
	PI vs. GI	55.3246	0.798	0.001
	PI vs. SK	239.6948	0.9448	0.001
	GI vs. SK	232.8743	0.9433	0.001
Southern catfish	ST vs. PI	10.3927	0.366	0.001
	ST vs. GI	29.7509	0.623	0.001
	ST vs. SK	30.6986	0.6304	0.001
	PI vs. GI	69.7964	0.7949	0.001
	PI vs. SK	101.333	0.8492	0.001
	GI vs. SK	26.03295	0.5912	0.001

*The effects were evaluated using PERMANOVA test with adonis function (*F* and *R*^2^ value) based on Bray-Curtis distances.*

**FIGURE 5:**
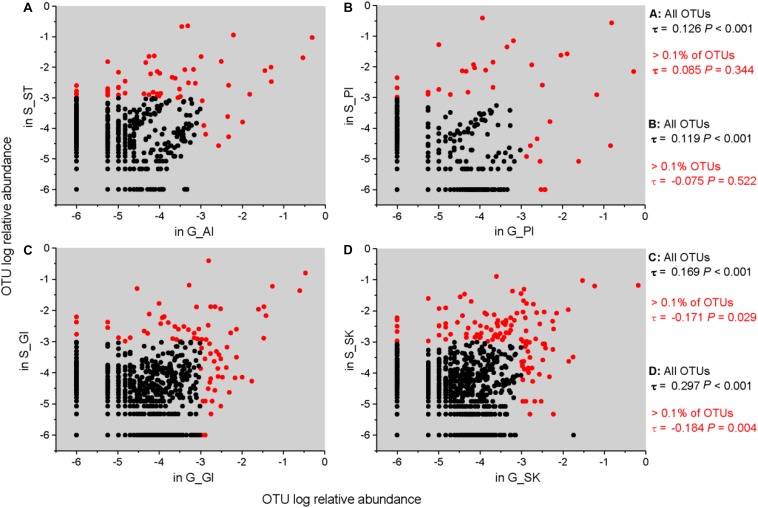
Correlations of relative abundance of microbial taxa in the same organs between grass carp and southern catfish at the OTU level. The correlations between G_AI and S_ST **(A)**, G_PI and S_PI **(B)**, G_GI and S_GI **(C)**, and G_SK and S_SK **(D)** are shown. To plot taxa absent in one host, a microsmall value was added to all frequencies before log transformation. Kendall’s ranked correlations, *τ* and *P*-values, are shown to the right of each plot.

We analyzed the correlation of relative abundances of OTUs between grass carp and southern catfish for the same body sites. The results showed that the abundance of all OTUs in grass carp was significantly and positively correlated with that in southern catfish for each body site (Figure 5), with the highest correlation value found on fish skin (stomach/anterior intestine, ST/AI: *τ* = 0.126, *P* < 0.001; posterior intestine, PI: *τ* = 0.119, *P* < 0.001; gill, GI: *τ* = 0.169, *P* < 0.001; skin, SK: *τ* = 0.297, *P* < 0.001). Unexpectedly, when more than 0.1% of OTU abundances were used for the correlative calculations, the correlations between grass carp and southern catfish were insignificant for both stomach/AI and PI (ST/AI: *τ* = 0.085, *P* = 0.344; PI: *τ* = − 0.075, *P* < 0.522). The correlations between the two fish species were significant but negative for gill and skin (GI: *τ* = −0.171, *P* < 0.029; SK: *τ* = − 0.184, *P* < 0.004). The direct comparisons showed no correlations of the most abundant OTUs present in internal body sites (gastrointestinal tract) between grass carp and southern catfish and an inverse correlation of those in external body sites (gill or skin) of the fish (Figure 5).

Alpha diversity differed significantly among body sites for both grass carp and southern catfish (ANOVA, *P* < 0.001 for all in grass carp and *P* < 0.001 for Shannon and *P* < 0.01 for observed species, Chao1, and ACE in southern catfish) (Figure 3), with the highest alpha diversity in gill of grass carp and that in skin of southern catfish. In grass carp, no differences were observed in the digestive tract (*P* > 0.05). In southern catfish, however, the stomach had markedly higher values than PI (*P* < 0.001 for all). All alpha diversity indexes but observed species in grass carp were higher in skin than gill (*P* < 0.05 for Shannon, Chao1, and ACE and *P* = 0.439 for observed species). In contrast, the significantly higher values in southern catfish were observed in the gill (*P* < 0.001 for all).

### Taxa in the Environment

**FIGURE 6:**
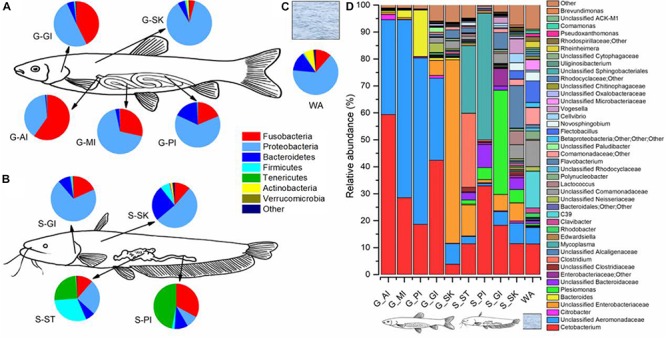
Microbiome community compositions of grass carp, southern catfish, and their rearing water. The microbial compositions of grass carp **(A)**, southern catfish **(B)**, and rearing water **(C)** at the phylum level; the microbial compositions of the two co-cultured fish and the rearing water **(D)** at the genus level.

To identify taxa that were enriched or depleted in the cohabiting fish and their environment, we examined the abundances of taxonomic compositions (Figure 6). In the rearing water, four bacterial phyla, Proteobacteria, Bacteroidetes, Fusobacteria, and Actinobacteria dominated 64.9, 14.0, 11.4, and 6.7% of the microbiome community, respectively (Figure 6C). At the genus level, there were 367 genera, with the abundances of 63 genera exceeding 0.1%. Of them, *C39* (13.6%), *Cetobacterium* (11.3%), unclassified Comamonadaceae (9.4%), *Flectobacillus* (8.0%), other unclassified Comamonadaceae (6.4%), and unclassified Aeromonadaceae (6.1%) were the most abundant (Figure 6D).

### Taxa of External Body Sites

In the gill of grass carp, the most dominant were the phyla Proteobacteria (50.6%), Fusobacteria (42.5%), and Bacteroidetes (5.0%), whereas they accounted for 70.4, 18.3, and 9.1% of the gill microbiome in southern catfish, respectively (Figures 6A,B). The three phyla dominated in the skin, followed by Firmicutes, but the abundances differed between grass carp and southern catfish (87.6 vs. 52.3% for Proteobacteria, 5.1 vs. 25.6% for Bacteroidetes, 4.1 vs. 1.5% for Fusobacteria, 1.1 vs. 6.8% for Firmicutes). In total, 367 genera were detected in gill and 420 in skin of grass carp, and 328 in gill and 409 in skin of southern catfish. The most dominant genera of external body sites were *Cetobacterium*, *Plesiomonas*, *Flavobacterium*, and some unclassified taxa from Aeromonadaceae, Enterobacteriaceae, Bacteriodaceae, Comamonadaceae, and Neisseriaceae (Figure 6D).

### Taxa of Internal Body Sites

We further investigated the microbial abundance of internal body sites. In grass carp, the phyla Fusobacteria and Proteobacteria dominated, followed by Bacteroidetes, with significant taxonomic decreases in Fusobacteria (59.7 to 18.6%) and increases in Proteobacteria (38.9 to 63.3%) from AI to PI (Figures 6A,B). The most significant shifts can be contributed to Bacteroidetes, with its abundance increasing from 0.9% in AI to 2.9% in medium intestine (MI) and to 17.4% in PI. Gastrointestinal tract microbiome of southern catfish was mainly composed of Tenericutes and Fusobacteria, whose relative abundances were lower in stomach (25.1 and 11.4%) than intestine (47.0 and 32.9%), while significantly higher abundances of Firmicutes (30.2 vs. 1.9%) and Proteobacteria (25.4 vs 8.8%) were observed in stomach compared to intestine. The differences were further reflected in multiple abundant lower taxa (Figure 6D). In grass carp, 200 genera in AI, 199 in MI, and 134 in PI were detected, whereas 411 in stomach and 107 in intestine in southern catfish.

## Discussion

Animals suffer the selective pressures of their habitat, eventually making necessary adaptation and/or evolvement for existence. In this study, we find that the microbiome of the cohabiting grass carp and southern catfish significantly differs from that of their rearing water and that fish microbiome is dependent on host species. The systematic ecological investigation of multiple organs disclosed taxonomic communities of microbiome between internal organs that are more similar than those between external organs in both fish species. Most important, the results reveal a separation pattern of microbiome among organs within host species, suggesting niche differentiation of the microbiome at the organ scale. Our findings, especially the considerations of multiple organs beyond the fish gut, provide vital information on understanding the microbiome assemblages of farmed fish.

### Divergences of Microbiome Between Fish and Their Environment

Host microbiome can be generally described as two kinds of microbial sources, autochthonous and allochthonous: the former is associated with the host and relatively permanent, whereas the latter is related with the food and rearing water. Herein, we can neglect the effect of microbial community of food on fish microbiome due to no microbes detected in the supplied feed. The shared OTUs by fish and their surrounding water were found, but the predominant microbes not only in internal organs but also in external organs were significantly different from those in the water, as also shown by Schmidt et al. (2015). Moreover, aquatic environment contains a higher microbial diversity (Chiarello et al., 2015). These suggest that the dominated microbial taxa in the two fish species are largely not dependent on the composition and abundance of microbial community in their aquatic environment and compete within host microenvironments to assemble their unique communities. The most abundant genus *C39* in the rearing water was rarely found in fish-related microbiome. By contrast, *Cetobacterium* is enriched in the gastrointestinal tract of many freshwater fish (Zhang et al., 2017), possibly leading to the occurrence in the water due to the flowing feces excretion into surrounding water (Pratte et al., 2018; Reinhart et al., 2019). It is supposed that *Cetobacterium* of the gill and skin of both grass carp and southern catfish might come from the digestive tract and is transferred to the water. In recent reef fish study, the presence of *Cetobacterium* in fish gill is accompanied with that in fish gut, and with low abundant *Cetobacterium* in their free-living environment (Pratte et al., 2018). Host-associated microbes might easily transfer and colonize body sites than microbial transfer between the fish and their environment.

### Host-Mediated Microbiome

Gut microbiome of vertebrates spanning humans, other mammals, and metazoan seems to be dependent on host phylogeny and morphology and their diet category (Ley et al., 2008). Microbes inhabiting fish may tightly interact with and further adapt to the host so it is likely that fish-associated microbiome is assembled (Chiarello et al., 2018). The host-associated samples within grass carp and southern catfish formed two distinct clusters, directly adding strong evidence of host-specific differences in microbiome. A site-to-site comparison of the samples collected from the two fish showed some similarities of the microbial composition and abundance at the whole OTU levels. When removing the low abundant OTUs, the correlations disappeared between internal organs and were negative between external organs, suggesting that the high abundant microbial taxa in grass carp tended to be lower in southern catfish and vice versa. The changes explained the similarities of most low abundant OTUs in the corresponding organs between grass carp and southern catfish. Recently, Baldo et al. (2017) compared gut microbiome of 29 cichlid species from two distinct lakes across a broad dietary and phylogenetic range, revealing clustering of gut microbiota largely following the dietary habits, yet not by host phylogeny. Although host factors are vital in shaping gut microbiome (Pearce et al., 2017), it is possible that host effects are outweighed by other confounding factors (Falony et al., 2016).

### Body Site-Driven Microbiome

Fish body sites such as digestive tract, gill, and skin evolve to permit colonization on the mucosal surfaces by complex commensal microorganisms (Gomez et al., 2013; Lowrey et al., 2015). The microbial partitioning of the co-cultured fish from a small ecological zone, such as a lab tank in this study, indicates negligible influences of habitat regions on external microbiome. Regardless of the technique used for the identification of external microbiome, to date, a handful of studies are involved in fish microbiome across body sites (Wang et al., 2010; Chiarello et al., 2015) but reporting highly different diversities of skin-associated microbial communities. This is supported by the results from external body sites between grass carp and southern catfish. Additional efforts on Pacific oysters displayed an initial establishment of microbial succession in organs-specific context to understand the interplay of microbes with a newly encountered environment (Lokmer et al., 2016), further providing insight into how fish transfer and polyculture affect their microbiome in aquaculture in that the practices involve initial onsets and adaptations of host microbes. Fish external organs are dominated by phylum Proteobacteria (Larsen et al., 2013; Chiarello et al., 2015; Mohammed and Arias, 2015), reflecting the advantage of unique niches for favoring microbial growth. This has been proven by the predominance of Proteobacteria in the skin of healthy salmonids and by the decreased abundance in individuals with an infection of salmonid alphavirus (Reid et al., 2017). Some nitrogen cycle symbionts from Proteobacteria that exert on the functional role of the microbial colonization on fish health in the gill can effectively convert harmful ammonia excreted by the host into harmless nitrogen gas (van Kessel et al., 2016). Therefore, it is critical to examine the external microbiome of more fish species to understand microbiome-associated host phylogenetic relatedness with their fitness and differences in environment tolerance and disease resistance. In such way, we could establish predictive models in microbial communities of economically important commercial fish species, modeling a proactive prevention of external microbes from getting out of control and causing illness.

In aquaculture, fish species co-cultured for improving utilizations of feed resources and space in ponds often have different digestive properties, and the feeds used in practice are mainly dependent on one of those fish species. Surprisingly, despite the long digestive tract of herbivorous grass carp, we found no differences in microbial diversity from anterior, to medium, and to posterior gut. This might be caused by the high-protein feed only used in this study, which does not meet the inherent feeding biology of herbivorous grass carp. Conversely, the differences in the relative short gastrointestinal tract of carnivorous southern fish are consistent with our previous observation (Zhang et al., 2017). The artificial feed containing no microbes in this study further excludes the possibility of the generalist microbial immigrants from food to the stomach compared to fresh feed supplied in the previous study. Stomach environment, although more rigorous and diverse, is a complex niche, harboring a more universal and heterogeneous microbial community. Fish in this study were sampled at two time points; however, they only were co-cultured in one tank. Further studies therefore are needed to generalize these results.

When a grass diet was replaced by an extreme animal diet for several days, Fusobacteria, with *Cetobacterium somerae* dominating the phylum, increased significantly in grass carp gut (Hao et al., 2017). In this study, we also found the high abundant Fusobacteria in grass carp by using a high-protein feed. One explanation could be attributed to dietary high protein: the genome of *Cetobacterium* (dominated by *C. somerae*) contains many functional gene families associated with protein digestion that supports the host response to the dietary change (Hao et al., 2017). The taxonomic enrichment might confer advantages to the host in defense against dietary stress. The clear gradient differences in microbial assemblages in the digestive tract further reflect niches differentiation at the organ scale (Ye et al., 2014; Gajardo et al., 2016; Zhang et al., 2017).

## Conclusion

This study presents a rare example of distinct microbiome of internal and external organs from two different biological traits of cohabitating fish species and indicates the thoroughly differential microbial associations with their living environment, indicating a crucial role of host selection in shaping the internal and external microbiome. Both grass carp and southern catfish simultaneously exhibited diverse microbiome community signatures in multiple organs, strongly supporting the hypothesis of body site-driven microbiome at the organ scale. The nature of these microbial differences, including those relevant to food digestion and mucosal immunity, remains to be tested. In practice, several lines of inquiry might be particularly warranted: extending these findings to polyculture fish species in aquaculture, coupled with the characteristics of their ecological niche, metabolic difference, and disease resistance, will help elucidate species specificity and variability of microbiome and further provide new insights into the functionality and causality of these communities in farmed fish.

## Data Availability Statement

The datasets generated for this study can be found in the PRJNA518052.

## Ethics Statement

The animal study was reviewed and approved by the Scientific Ethics Committee of Huazhong Agricultural University.

## Author Contributions

ZZ and DL designed the experiment and analyzed the data. ZZ, WX, and RT conducted the experiment. ZZ wrote the manuscript. DL, RT, and LL revised the manuscript.

## Conflict of Interest

The authors declare that the research was conducted in the absence of any commercial or financial relationships that could be construed as a potential conflict of interest.
