# Supplementary material for: Microbiome of Co-cultured Fish Exhibits Host Selection and Niche Differentiation at the Organ Scale
Source: Front Microbiol. 2019 Nov 8;10:2576. doi: 10.3389/fmicb.2019.02576 (PMC6856212; doi:10.3389/fmicb.2019.02576)
Supplement: Supplementary file 1 [file Data_Sheet_1.PDF]

Supplementary materials

Microbiome of co-cultured fish exhibits host selection and niche differentiation at the organ scale

Zhimin Zhang[1,2], Dapeng Li[1,*], Weitong Xu[1], Rong Tang[1], Li Li[1]

[1]College of Fisheries, Hubei Provincial Engineering Laboratory for Pond Aquaculture, Huazhong Agricultural University, Wuhan 430070, China

[2]Institute of Hydrobiology, Chinese Academy of Sciences, Wuhan 430072, China

Table S1. The parameters of rearing water of grass carp and southern catfish in the tank.

Sampling time	T (℃)	DO (mg/l)	pH	NO_3^- (mg/l)	NO_2^- (mg/l)	TAN (mg/l)
42 days	27.5	6.74	7.83	11.05	0.23	0.27
45 days	27.3	6.67	7.79	9.87	0.16	0.31

Table S2. The body weight of grass carp and southern catfish.

Species	Body weight (g)		
	0 days	42 days	45 days
Grass carp	128.9	259.5	251.3
Southern catfish	19.1	182.8	189.6

Table S3. The effect of fish species and body sites on microbial community structure.

Source	d.f.	SS	MS	*F*-Model	R^2	*P value*
Species	1	5.4417	5.4417	56.47	0.2735	0.001
Body sites	5	7.4204	1.4841	15.401	0.37295	0.001
Residual	73	7.0346	0.0964		0.35356	
Total	79	0.7504				
